# Functional categorization of unique expressed sequence tags obtained from the yeast-like growth phase of the elm pathogen *Ophiostoma novo-ulmi*

**DOI:** 10.1186/1471-2164-12-431

**Published:** 2011-08-24

**Authors:** William Hintz, Michael Pinchback, Paul de la Bastide, Steven Burgess, Volker Jacobi, Richard Hamelin, Colette Breuil, Louis Bernier

**Affiliations:** 1 Biology Department, University of Victoria, P.O. Box 3020 STN CSC, Victoria, BC, V8W 3N5, Canada; 2 Centre d'étude de la forêt (CEF), Faculté de foresterie et de géomatique, Université Laval, Québec (Québec) G1K 7P4, Canada; 3 Service canadien des forêts, Ressources naturelles Canada, Centre de foresterie des Laurentides, 1055 du PEPS, P.O. Box 3800, Québec (Québec) G1V 4C7 Canada; 4 Department of Wood Science, University of British Columbia, Vancouver, British Columbia, V6T 1Z4 Canada

## Abstract

**Background:**

The highly aggressive pathogenic fungus *Ophiostoma novo-ulmi *continues to be a serious threat to the American elm (*Ulmus americana*) in North America. Extensive studies have been conducted in North America to understand the mechanisms of virulence of this introduced pathogen and its evolving population structure, with a view to identifying potential strategies for the control of Dutch elm disease. As part of a larger study to examine the genomes of economically important *Ophiostoma *spp. and the genetic basis of virulence, we have constructed an expressed sequence tag (EST) library using total RNA extracted from the yeast-like growth phase of *O. novo-ulmi *(isolate H327).

**Results:**

A total of 4,386 readable EST sequences were annotated by determining their closest matches to known or theoretical sequences in public databases by BLASTX analysis. Searches matched 2,093 sequences to entries found in Genbank, including 1,761 matches with known proteins and 332 matches with unknown (hypothetical/predicted) proteins. Known proteins included a collection of 880 unique transcripts which were categorized to obtain a functional profile of the transcriptome and to evaluate physiological function. These assignments yielded 20 primary functional categories (FunCat), the largest including Metabolism (FunCat 01, 20.28% of total), Sub-cellular localization (70, 10.23%), Protein synthesis (12, 10.14%), Transcription (11, 8.27%), Biogenesis of cellular components (42, 8.15%), Cellular transport, facilitation and routes (20, 6.08%), Classification unresolved (98, 5.80%), Cell rescue, defence and virulence (32, 5.31%) and the unclassified category, or known sequences of unknown metabolic function (99, 7.5%). A list of specific transcripts of interest was compiled to initiate an evaluation of their impact upon strain virulence in subsequent studies.

**Conclusions:**

This is the first large-scale study of the *O. novo-ulmi *transcriptome. The expression profile obtained from the yeast-like growth phase of this species will facilitate a multigenic approach to gene expression studies to assess their role in the determination of pathogenicity for this species. The identification and evaluation of gene targets in such studies will be a prerequisite to the development of biological control strategies for this pathogen.

## Background

Throughout the twentieth century, the American elm (*Ulmus americana*) has been a favoured urban tree for planners and landscape architects in many North American cities, providing shade along innumerable streets and boulevards. The elm is a particularly popular choice in northern climates because of its resistance to extremes of weather and harsh urban growing conditions, while its abundant crown foliage is large enough to span a city street [1]. Unfortunately, populations of this urban tree have been decimated by Dutch elm disease. The disease in North America can be attributed to two separate introduction events: the early epidemic caused by the non-aggressive sub-group *O. ulmi *and the later, more severe epidemic, caused by the highly pathogenic aggressive sub-group of *O. novo-ulmi*, which continues to threaten elm populations of Western Canada.

Genomic fingerprinting methods are useful for resolving phylogenetic relationships among closely related populations and species [2] and for the reconstruction of population histories, especially for a species introduction, where there can be rapid population development [3]. Isolates of *O. novo-ulmi *sampled across Saskatchewan and Manitoba were analyzed using both nuclear and mitochondrial genetic markers and only limited genetic variability was detected. All of the isolates represented the aggressive sub-group and included only two distinct nuclear and four mitochondrial genotypes [4]. The vast majority of isolates were of a single genotype, suggesting that one genetic individual dominated the sample area. Later analysis in the same region compared isolates collected in 1993 and 2002, using both RAPD markers and an evaluation of vegetative compatibility (vc) [5]. It was hypothesized that new vc types would develop quickly after the disease front had passed through the region [6,7]. Compatibility tests confirmed a single vc group, demonstrating that a genetically uniform population persists in western Canada. In contrast, a much greater diversity of vc types has been documented in the Eurasian aggressive (EAN) race of *O. novo-ulmi*, as compared to populations of the North American aggressive (NAN) race [6]; the EAN and NAN subpopulations of *O. novo-ulmi *have since been re-designated as subspecies *novo-ulmi *and *americana*, respectively [8]. A low diversity of vc types for the *americana *subspecies appears to be concentrated in the southern Great Lakes, which is consistent with its initial detection in this region; areas colonized more recently, including western Canada, display very limited vc diversity [6,8,9]. In areas of Europe experiencing a well established epidemic of subspecies *novo-ulmi *that was initially characterized by a uniformity of vc types, vegetative incompatibilities have been reported within six to ten years [9]. In contrast, the comparatively low diversity of vc groups observed for the subspecies *americana *is atypical of an established pathogen epidemic, although rapidly expanding pathogen populations have previously been reported to exhibit low genetic diversity [10].

Factors influencing the development of vc groups and increased genetic diversity in subspecies *novo-ulmi *must therefore be significantly different from those encountered by subspecies *americana*. There is no clear explanation for the limited genetic variability observed in the *O. novo-ulmi *subspecies *americana *population in western Canada. The report of only two nuclear genotypes, and no transitional genotypes, suggests that sexual events are rare and that its propagation has been predominantly by asexual means within the time frame of this epidemic [4,5]. In a previous study of North America populations of this species, two possible factors contributing to low vc diversity were suggested: the infrequent occurrence of deleterious d-factor viruses in populations provide a low level of selection for new vc types and the frequent predominance of single vc clones on a host substrate does not favour the establishment of novel vc types [9]. The role of host genetic diversity has not been evaluated to any extent in studies of Dutch elm disease and it should be noted that surveys of elm populations in western Canada have been conducted primarily in urban environments and may thus have favoured planted nursery stocks of this species. This may represent a more limited diversity compared to wild *U. americana *trees.

From a perspective of disease management, the genetic uniformity of the subspecies *americana *population could be exploited as a target for the control of Dutch elm disease in western Canada through the use of fungal hypoviruses and related genetic tools to reduce pathogen virulence [6]. The presence of double-stranded RNA (dsRNA) viruses in isolates of *O. novo-ulmi *has been well-documented [5,11,12] and may play a role in strain fitness and the genetic diversity of the pathogen, including the diversity of vc types [9,13,14]. Extensive studies have been done to understand the mechanisms of virus-determined hypovirulence observed in the causal agent of chestnut blight, *Cryphonectria parasitica*, and to establish its utility as a method of disease control for the North American tree species American chestnut (*Castanea dentata*)[15-17]. Similarly, the introduced ascomycete *O. novo-ulmi *has become a serious pathogen of a major tree species and represents a good candidate for virus-mediated control.

Until recently, there has been little work on profiling gene expression in *O. novo-ulmi*. A study focused on the transcriptome represents an opportunity for extensive gene discovery. The primary benefit of this approach is the detection and assessment of genes potentially implicated in pathogenicity and parasitic fitness. Wound pathogens, such as *O. novo-ulmi*, directly enter the host through a pre-existing wound. *Ophiostoma novo-ulmi *is a dimorphic fungus, alternating between a budding yeast-like growth form and a filamentous growth form, and this morphology switch appears to have great significance to pathogenicity [18]. The yeast phase has been proposed to be involved in dissemination of the pathogen from tree to tree by the insect vector as well as translocation of the infection within the host tree [19]. The mycelial form is required to penetrate from one vessel to another and may thus be considered the invasive form [18]. The yeast - hyphal transition is regulated by environmental factors and occurs in the homokaryotic (haploid) state [19]. The cataloguing and functional categorization of a library of expressed sequence tags (ESTs) from the yeast form of this fungus provides a means of identifying genes integral to the first stages of infection. A more complete understanding of the genetic basis of pathogenicity could provide targets for gene regulation, leading to methods of disease control The recent demonstration of targeted gene disruption in *O. novo-ulmi *by RNA interference [20], combined with knowledge about specific target genes as detected by EST analysis, makes this goal more readily achievable.

The Canadian *Ophiostoma *Genome Project was first initiated in 2001 as a collaborative effort with the general objective of the large-scale collection and analysis of genome data for species of this genus [21]. Longer-term studies will include the examination of specific genes that are differentially expressed, especially those that relate to mechanisms of pathogenicity in these species. The objectives of the current study were to (i) construct a low-redundancy EST library using total RNA extracted from the yeast-like growth phase of isolate H327 of *Ophiostoma novo-ulmi*, (ii) annotate the EST information by determining their closest matches to known or theoretical sequences in public databases, and (iii) categorize the known EST collection to obtain a functional profile of the *O. novo-ulmi *genome, as expressed under these conditions of growth. This work will eventually be assisted by the construction of an EST microarray or RNA Seq analysis to facilitate genome-level studies of gene expression.

## Results

### Sequencing of library and BLASTX analysis

Analysis of novel sequence data typically begins with the assignment of putative identities based on alignments with derived proteins in public databases [20]. Recent genome sequencing projects have resulted in the deposition of hundreds of thousands of theoretical proteins, predicted by analysis of sequenced genomes. Theoretical proteins frequently match with novel ESTs at a high alignment score, but are of little consequence if they do not assign function or identity to the EST. A protein of known function or identity will provide more meaningful information, even at a lesser alignment score. While automated alignment and annotation algorithms serve to provide a good approximation of most EST identities, manual scrutiny and annotation is necessary to improve fidelity. With these constraints in mind, we began an analysis of the expressed sequences of the Dutch elm pathogen *O. novo-ulmi*.

The DNA sequence was determined for 5,760 clones of a library that was estimated to contain a total of 22,000 clones. The proportion of unique sequences identified in the entire yeast LMW library gradually declined as sequencing progressed, but remained above 30% of all sequences read within the final 96-well cell culture plate. This suggests that there still remains a sizable resource of unique *O. novo-ulmi *sequences in the cDNA library.

Library data is summarized in Table 1. Of the 5,760 EST clones sequenced, 4,386 gave readable sequence information (~ 76%) and included inserts ranging from 133 to 690 bp with an average insert size of 498 bp. A total of 2,093 of the 4,386 readable sequences matched entries described in NCBI and GenBank public databases, as determined by BLASTX analysis [22]. These included 1,761 matches with known proteins and 332 matches with unknown (hypothetical/predicted) proteins. Matches with known proteins included 880 unique transcripts corresponding to 49.97% of the EST sequences in this category. Applying this same ratio to the category of unknown proteins would generate an additional 166 unique transcripts among this group, for a total of 1,046 single matched sequences.

**Table 1:** Summary of EST sequence analysis for the *O.novo-ulmi *Yeast LMW library.

Parameter	Number
Total ESTs sequenced	5,760
Average length of EST (bp)	498
Readable sequence data	4,386
Sequences matching public databases	2,093
Sequences matching known proteins	1,761
Sequences matching unknown (hypothetical/predicted) proteins	332
Sequences matching known, unique proteins (singletons)	880
Sequences matching unknown proteins in public databases that represent singletons (estimated as 50% of unknown proteins)	166
Total of singletons matching known and unknown unique proteins	1,046
Sequences with no matches in public databases	2,293
Sequences with no matches in public databases (less 20% containing non-authentic sequences)	1,835
Sequences with no matches in public databases that represent singletons (estimated as 50% of unmatched)	917
Total unique sequences from all categories	1,963

A total of 2,293 of the 4,386 readable sequences drew no matches by BLASTX analysis. It may be assumed that 20% of these clones contained non-authentic sequences, due to the ligation of random fragments of DNA into vectors during the creation of the EST library, thus reducing the total to 1,835 sequences without a match. Based on the results for matched readable sequences, it was estimated that approximately 50% of unmatched EST sequences were unique, thus yielding an additional 917 sequences that are at present unidentified. The total number of unique sequences from all categories is therefore estimated to be 1,963 (880 known proteins, 166 unknown proteins and 917 unmatched sequences). Given that the *O. novo-ulmi *genome is estimated to contain 8,000 - 10,000 genes [23,24], the total number of unique sequences in this library is estimated to represent about 22% of this genome. Additional sequencing of EST library clones will add further depth to this analysis.

### Functional assignment of ESTs

Functional assignment of expressed sequences requires a consideration of the metabolic pathway in which a gene product is likely to be active. In some instances, the presence of a characteristic functional group or structural domain indicates the probable molecular mechanism of a protein, but offers no insight into the physiological function that protein serves [25,26]. While the specific molecular mechanism of a specific protein may be known, inferences regarding the physiological role of similar proteins can be made based on their conservation of consensus sequences [25]. Sequences involved in target-ligand interactions are often similar among related proteins and provide a means of deducing their putative physiological role by comparison with previously categorized proteins bearing similar consensus sequences. The 880 matched unique transcripts were selected as a subset of the 5,760 EST fragments and subjected to further BLAST analysis to obtain the three highest scoring alignments. These data were manually scrutinized and each EST was manually annotated using the FunCat system. A summary of results for the unique transcripts is provided in Additional File 1.

### Functional assignment of O. novo-ulmi yeast LMW ESTs to primary categories

The assignment of the *O. novo-ulmi *Yeast LMW ESTs with known identities (n = 880) into functionally related groups yielded 20 primary functional categories (Table 2). The unclassified category (99, 7.5% of total) represented ESTs for which a protein identity could be assigned based upon an alignment with known sequences, but the metabolic function of that sequence remained unknown. The largest categories for the functional assignment of ESTs of known function included (in order of decreasing importance) Metabolism (01, 20.28%), Protein synthesis (12, 10.14%), Sub-cellular localization (70, 10.23%), Biogenesis of cellular components (42, 8.15%) and Transcription (11, 8.27%). Individual categories that represented less than 1.0% of total assignments included Protein fate (14, 0.85%), Protein activity regulation, (18, 0.82%), Cell cycle and DNA processing (10, 0.7%), Transposable elements, viral and plasmid proteins (38, 0.34%), Interaction with the environment (36, 0.25%), Cell fate (40, 0.23%) and Cell type differentiation (43, 0.11%).

**Table 2:** Classification of ESTs by functional category.

Classification by functional category^1^	Representation by EST fragments^2^	Percent of total Representation^3^
01 Metabolism	178.5	20.28
02 Energy	39.5	4.49
10 Cell cycle and DNA processing	6	0.7
11 Transcription	72.75	8.27
12 Protein synthesis	89.25	10.14
14 Protein fate	7.5	0.85
16 Protein with binding function or co-factor requirement	12.25	1.39
18 Protein activity regulation	7.25	0.82
20 Cellular transport, facilitation and routes	53.5	6.08
32 Cell rescue, defence and virulence	46.75	5.31
34 Interaction with the cellular environment	32	3.64
36 Interaction with the environment	2.16	0.25
38 Transposable elements, viral and plasmid proteins	3.0	0.34
40 Cell fate	2.0	0.23
42 Biogenesis of cellular components	71.75	8.15
43 Cell type differentiation	1.0	0.11
70 Subcellular localization	90	10.23
73 Cell type localization	30	3.41
98 Classification unresolved	51	5.80
99 Unclassified proteins	66	7.5
Subtotal =	862.25	97.98
All remaining categories (each representing < 0.1% of total)	17.75	2.02
Total =	880	100.00

Assignment of EST fragments by functional category and the percent representation of each category in the collection of the *O. novo-ulmi *yeast LMW library.

^1^Based upon the MIPS classification scheme for the functional annotation of protein sequences [50].

^2^Classification of known yeast LMW sequences, as determined by BLASTX searches and homology to sequences of known identity.

^3^Relative percentage of known yeast LMW sequences in each functional category.

### Functional assignment of O. novo-ulmi yeast LMW ESTs to subcategories

Each of the eight primary functional categories that represented more than 4.5% of all identified ESTs were categorized to the secondary level within each category (Table 3). The subcategories represented in each group exhibited a wide variation in both the number detected and in the proportional distribution among these subcategories. FunCat 99 (Unclassified proteins, 7.5%) represented 66 standardized functional assignments of ESTs.

**Table 3:** Distribution of identified ESTs within each of the primary functional categories.

Functional Category	Functional Subcategory	Percent occurrence
01 Metabolism	01.01 amino acid metabolism	19.61
	01.02 nitrogen and sulfur metabolism	5.60
	01.03 nucleotide metabolism	11.76
	01.04 phosphate metabolism	2.10
	01.05 C-compound and carbohydrate metabolism	29.83
	01.06 lipid, fatty acid and isoprenoid metabolism	23.53
	01.07 metabolism of vitamins, cofactors, and prosthetic groups	4.48
	01.20 secondary metabolism	3.08
02 Energy	02.07 pentose-phosphate pathway	0.63
	02.10 tricarboxylic-acid pathway (citrate, Krebs and TCA cycles)	9.43
	02.11 elect. trans. and mem.-associated energy conservation	2.52
	02.13 respiration	60.38
	02.16 fermentation	11.32
	02.19 metabolism of energy reserves (e.g. glycogen, trehalose)	5.03
	02.45 energy conversion and regeneration	10.69
11 Transcription	11.02 RNA synthesis	86.25
	11.04 RNA processing	11.00
	11.06 RNA modification	2.75
12 Protein synthesis	12.01 ribosome biogenesis	77.31
	12.04 translation	20.45
	12.10 aminoacyl-tRNA-synthetases	2.24
20 Cellular transport, transport facilitation and transport routes	20.01 transported compounds (substrates)	39.27
	20.03 transport facilitation	11.42
	20.09 transport routes	49.32
32 Cellular rescue, defense and virulence	32.01 stress response	43.32
	32.05 disease, virulence and defense	15.51
	32.07 detoxification	41.18
42 Biogenesis of cellular components	42.01 cell wall	29.97
	42.02 eukaryotic plasma membrane	1.39
	42.03 cytoplasm	40.77
	42.04 cytoskeleton	7.67
	42.07 endoplasmic reticulum	0.35
	42.10 nucleus	3.14
	42.16 mitochondrion	9.76
	42.19 peroxisome	3.83
	42.25 vacuole or lysosome	1.74
	42.27 extracellular/secretion proteins	1.39
70 Subcellular localization	70.01 cell wall	12.36
	70.02 eukaryotic plasma membrane/membrane attached	20.51
	70.03 cytoplasm	30.90
	70.04 cytoskeleton	1.12
	70.07 endoplasmic reticulum	23.88
	70.10 nucleus	11.24
	70.25 vacuole or lysosome	1.12

The FunCat 01 (Metabolism) was comprised of 178.5 standardized functional assignments of identified ESTs, making it the most highly represented functional category. Within this primary category, eight subcategories relating to metabolism were represented. Expressed sequence tags associated with carbon compound metabolism (01.05) were the most highly represented, comprising 29.83% of FunCat 01. Enzymes implicated in the metabolism of fatty acids (01.06) and amino acids (01.01) were also highly represented, comprising 23.53% and 19.61%, respectively, of these subcategories. The functional assignment of ESTs associated with nucleotide metabolism (01.03) were also important (11.76%). The remaining subcategories represented the metabolism of nitrogen and sulphur (01.02), phosphate (01.04), vitamins, cofactors and prosthetic groups (01.07), and secondary metabolism (01.20), each of which comprised 5.6% or less of all subcategories.

A total of 39.5 standardized functional assignments and seven subcategories were represented within FunCat 02 (Energy), with the vast majority of ESTs occurring in the respiration (02.13) category (60.38%), followed by fermentation (02.16, 11.32%) and energy conversion and regeneration (02.45, 10.69%). The TCA cycle (02.10) was also well-represented (9.43%). Those subcategories exhibiting the least representation within FunCat 02 included ESTs classified within metabolism of energy reserves (02.19, 5.03%), electron transport and membrane-associated energy conservation (02.11, 2.52%), and the pentose-phosphate pathway (02.07, 0.63%).

Of the eight primary functional categories examined at the secondary level, FunCat 11 (Transcription) exhibited the least complexity, with 72.75 standardized functional assignments of ESTs in three subcategories, primarily RNA synthesis (11.02, 86.25%), RNA processing (11.04, 11.00%) and RNA modification (11.06, 2.75%).

The expression profile for FunCat 12 (Protein synthesis) had a similar distribution of functional assignments, with ribosome biogenesis representing the largest subcategory (12.01, 77.31%), followed by translation (12.04, 20.45%) and aminoacyl-tRNA synthetases (12.10, 2.24%), with a total of 89.25 standardized functional assignments. Genes in the largest subcategory were dominated by 40S and 60S ribosomal proteins.

The assignment of ESTs to subcategories within FunCat 20 (Cellular transport) included 53.5 standardized functional assignments. This category was comprised of three subcategories, two of which were highly represented, transport routes (20.09, 49.32%), transported compounds (substrates) (20.01, 39.27%) and transport facilitation (20.03, 11.42%).

Within FunCat 32 (Cell rescue, defence and virulence) a total of 46.75 standardized functional assignments were made in three subcategories. The assignment of ESTs associated with stress response (32.01) and detoxification (32.07) were almost equally represented at 43.32% and 41.18%, respectively, followed by the subcategory of disease, virulence and defence (32.05, 15.51%). Those ESTs associated with stress response were represented by inducible gene products sensitive to environmental stimuli, such as UV irradiation, desiccation and heat shock.

The greatest number of subcategories was observed for FunCat 42 (Biogenesis of cellular components). A total of 71.75 standardized functional assignments were distributed among ten subcategories. Those ESTs associated with cytoplasm biogenesis represented the largest subcategory (42.03, 40.77%) and included a number of chitin synthase (42.03) proteins, of importance to cell wall biogenesis [27]. The cell wall subcategory was the next largest group (42.01, 29.97%) and included genes coding for beta-glucanase/beta-glucan synthetase (42.01), stomatin, mucin, and cell wall surface anchor family protein. Subcategories with fewer assignments included mitochondrion (42.16, 9.76%) and cytoskeleton (42.04, 7.67%) biogenesis. The six remaining subcategories, totalling 11.84%, included peroxisome (42.19, 3.83%), nucleus (42.10, 3.14%), vacuole or lysosome (42.25, 1.74%), extracellular/secretion proteins (42.27, 1.39%), plasma membrane (42.02, 1.39%), and endoplasmic reticulum (42.07, 0.35%) biogenesis. In the peroxisome subcategory, the Woronin body major protein (42.19) was identified and is known to be important to cellular integrity during growth [28].

The subcategories of FunCat 70 (Subcellular localization) totalled 90 standardized functional assignments, that were distributed among seven subcategories. The greatest proportion of ESTs were associated with cytoplasm localization (70.03, 30.90%). Other main subcategories included ESTs associated with endoplasmic reticulum localization (70.07, 23.88%) and plasma membrane/membrane attached subcellular localization (70.02, 20.51%). The subcategories of cell wall (70.01, 12.36%) and nucleus (70.10, 11.24%) subcellular localization were also prominent, followed by genes coding for vacuole or lysosome (70.25) and cytoskeleton (70.04) localization that both comprised 1.12% of all assignments in each category.

The *O. novo-ulmi *unique transcript collection was reviewed and we identified a number of expressed genes that may be placed in these gene families of importance to ascomycetous pathogens (Table 4). Genes of interest included those relevant to cell wall biogenesis, pathogen defense mechanisms during infection and the host infection process.

**Table 4:** Transcripts detected in *O.novo-ulmi *that occur in gene families described for other ascomycetous pathogens and may function in determining virulence and fitness.

Assigned protein identity	No. of copies	FunCat number	Putative function	Species occurrence of gene locus (citation)
Trichothecene C-15 hydroxylase	1	01.06	Mycotoxin pathway	FG, FS [51,52]
Isochorismatase family hydrolase	2	99	Response to plant defence mechanisms	MG, BC, SS, SN, AF [FGI, 53]
Woronin body major protein	3	42.19	Cellular integrity (seal septal pores in response to stress, important during host infection)	MG, filamentous ascomycetes [FGI, 54]
Tetraspanin	2	99	Transmembrane protein implicated in penetration of host tissue	BC, MG, CL [FGI, 55]
Cox17p, involved in copper metabolism and assembly of cytochrome oxidase	1	02.13; 70.10; 34.01	Cellular respiration (cytochrome C oxidase copper chaperone)	BC, MG, SS, SN, AN (FGI)
Beta-glucanase/ beta-glucan synthetase	1	01.05, 42.01	Cell wall biogenesis	BC, SS, SN, AN, FS [FGI, 56]
Copper-zinc superoxide dismutase	5	11.01; 11.07	Antioxidant defenses (conversion of superoxide radicals)	CN, PM [57]
Glutathione peroxidase paralogue	1	11.07	Antioxidant defenses (reduction of lipid hydroperoxides)	BC, MG, SS, SN, CG [FGI, 58]
Chitin synthase (including type A, C, 3, class IV and class V)	12	01.05, 34.11, 42.03, 73.01	Cell wall biogenesis	AN, AF, CGr, MG, PB [27]
Histidine kinase	1	14.07, 30.05	Global gene regulation	BD, HC [41]
Glucan synthase	1	01.05	Cell wall biogenesis	BD, HC, PB [58]

Species acronyms: AN = *Aspergillus nidulans*, AF = *Aspergillus flavus*, BC = *Botrytis cinerea*, BD = *Blastomyces dermatitidis*, CGl = *Chaetomium globosum*, CGr = *Colletotrichum graminicola*, CL = *Colletotrichum lindemuthianum*, CN = *Cryptococcus neoformans*, FG = *Fusarium graminearum *(anamorph of *Gibberella zeae*), FS = *Fusarium *spp., HC = *Histoplasma capsulatum*, MG = *Magnaporthe grisea*, PB = *Paracoccidioides braziliensis*, PM = *Penicillium marneffei*, SN = *Stagonospora nodorum*, SS = *Sclerotinia sclerotiorum*. FGI = Fungal Genome Initiative, Broad Institute http://www.broad.mit.edu/annotation/fungi/fgi/index.html

## Discussion

### Understanding pathogenicity in *O. novo-ulmi*

The construction of an EST library provides an initial gene expression profile for the yeast phase of a highly aggressive strain of the elm pathogen *O. novo-ulmi*. This EST library will be the first step in elucidating the complex mechanisms determining fungal pathogenicity, through the study of multiple candidate genes that are potentially implicated in the infection process. Historically, studies of pathogenicity were limited to one or a small number of candidate loci. With the creation of an EST library and the eventual use of microarray analysis to evaluate the expression of many genes under defined conditions, it will be possible to study whole organism gene expression as it relates to pathogenicity. The multigenic character of fungal pathogenicity can thence be more effectively assessed by this approach. Past efforts focused on single genes have attained limited success and have only confirmed the complex nature of fungal pathogenicity in *O. novo-ulmi *[29]. Information gained from future studies will be of benefit to understanding the elm pathogen, as well as other fungal pathogens of woody plant species.

### Comparision with other *Ophiostoma *species

The EST library will also serve as a comparative database for other studies underway in the *Ophiostoma *Genome Project for other growth states *O. novo-ulmi *and for other species of the genus *Ophiostoma *that target different hosts [30-34]. Associated data from the current project includes a total of 561 EST fragments (Genbank Acc: EG355614.1 to EG356175.1) from libraries that selected for perithecial (Onu-Per, 128 EST's), synnematal (Onu-Syn, 181), mycelium grown at 15°C (Onu-t15, 156) and mycelium grown at 31°C (Onu-t31, 96) growth phases [35]. The comparison of expressed sequences for different life phases will facilitate our preliminary analysis of differentially expressed genes in *O. novo-ulmi *and provide direction for future studies of genes relevant to pathogenesis. Existing EST projects for other *Ophiostoma *species include the sap-staining fungi *Ophiostoma piliferum *(Fungal Genomics Project, Concordia University, web address: https://fungalgenomics.concordia.ca/fungi/Opil.php), *Grosmannia clavigera *(formerly *Ophiostoma clavigerum*) [30,36,37] and *Ophiostoma floccosum *(Farrell et al., pers. com.)

The search for proteins associated with the pathogenic life phase of *Ophiostoma *spp. has produced various strategies designed to favour the expression of the relevant gene families. The use of suppressive subtractive hybridization PCR for the screening of genes differentially expressed in yeast and mycelia forms of the sap-stain fungus *Ophiostoma piceae *has demonstrated one strategy for the identification of genes involved in morphology switching [31]. More recently, an EST library was created for the lodgepole pine pathogen *G. clavigera*, using selective media to favour the detection of fungal genes expressed in the presence of oleoresin, one of the key host tree defense mechanisms against fungal pathogens [30]. This study described 5,974 EST fragments (2,600 unique transcripts) and their preliminary functional analysis was generally focused on those genes implicated in fungal growth within the host and pathogenicity. Similarly, an EST library for *O. piliferum *was constructed by culturing the fungus on different carbon sources to obtain a total of 9,589 EST fragments (Tsang, Storms and Butler, unpublished); this species has been considered for industrial applications, including the biopulping process [38]. Useful insights into gene families linked to virulence and growth within the host for *O. novo-ulmi *could be obtained by reviewing the EST data for *G. clavigera *and *O. piliferum*. Molecular mechanisms underlying Dutch elm disease were recently studied with the construction of an interaction cDNA library, by means of suppression subtractive hybridization from elm callus tissue following inoculation with *O. novo-ulmi*. Fifty three up-regulated Elm host-specific unique transcripts were identified, including genes coding for known classes of pathogenesis-related proteins [39].

### Strategies for detecting genes that influence virulence in *O. novo-ulmi*

The NCBI public database for submitted fungal EST sequences includes a total of 2,909,255 entries for 216 species, with 1,931,468 entries for 134 species of ascomycetes alone (November 2010). Among the ascomycetous species, there are a number of phytopathogens that have been the subject of genome sequencing projects, many of which are available in public databases [40]. In our efforts to indentify unique fungal genes relevant to pathogenicity, two general strategies have been followed in studies of *O. novo-ulmi*. We have considered other phytopathogenic ascomycete species as the most relevant group of organisms that may share common genes of importance to the host infection process, as well as dimorphic species of ascomycete pathogens that undergo radical changes in morphology upon host infection. A comparison of gene inventories for filamentous pathogenic and non-pathogenic ascomycetes identified a set of gene families that appear to have increased in diversity over evolutionary history and may play a role in pathogenicity [26]. Genes seen in phytopathogenic fungi are not necessarily unique to pathogen species, but have developed a greater diversity of related genes for specialized functions of a pathogenic lifestyle, when compared to homologues that are found in non-pathogenic species [26]. These specialized functions can include the production of secondary metabolites (mycotoxins, melanin, hydrophobins), the ability to use a variety of nutritional substrates, phenotypic plasticity (infection structures, dimorphism) and complex signalling pathways relevant to the infection process (host recognition, host defence systems, regulation of morphogenesis).

*Ophiostoma novo-ulmi *exhibits mycelial and yeast-like growth phases at different stages of growth and infection of the host elm. Possession of a variable growth phase is shared with some important human pathogenic fungi, where specific cues from the host species will induce the change in morphology. A multigenic approach has been pursued with these ascomycete pathogens and has begun to provide some important findings regarding the regulation of specific pathogen loci and the infection process [41,42]. A consideration of these genes in the screening of *O. novo-ulmi *library may therefore provide useful information. Histidine kinases in *Blastomyces dermatitidis *and *Histoplasma capsulatum *appear to act as global regulators in these dimorphic, human pathogenic ascomycetes, functioning in a two-component signalling system to regulate dimorphism and virulence. They directly influence the transition from mycelial to yeast phase in the body of a host and have been demonstrated to regulate the expression of several yeast-phase specific genes [41]. A single histidine kinase was identified in the EST library (FunCat 14.07, 30.05), providing a potential gene target for further evaluation. Also in *B. dermatitidis, H. capsulatum *and *Paracoccidioides braziliensis *the gene alpha-(1,3)-glucan synthase and several other loci are considered yeast-phase specific virulence genes, as they are up-regulated with the switch to the pathogenic yeast form at 37°C in the host [42]. In the species *H. capsulatum*, this is one of the genes regulated by a histidine kinase. The *O. novo-ulmi *library also contains glucan synthase (FunCat 01.05) and related genes that code for polysaccharides and other cell wall components.

A number of candidate virulence factors are under consideration for human pathogenic fungi and include melanin compounds, oxidative and nitrosative stress defense mechanisms, cell adhesion compounds, specific secreted products, arginine catabolism, cell surface composition, and those genes that are preferentially expressed in the parasitic yeast phase [42]. Since the transition of these dimorphic fungi from a mycelial to a yeast phase is required for virulence, this latter category has received much attention. For genomic studies of the species of *H. capsulatum *and *P. brasiliensis*, a large number of differentially expressed genes have been identified (500 and 328 genes, respectively) with the transition to the pathogenic yeast phase [43-45]. These genes fall into a number of functional categories and have provided a valuable resource for current studies of phase-specific gene expression in these species. Further study of the current yeast EST database created for *O. novo-ulmi *and its comparison to the EST library constructed for the mycelia growth phase of this species should allow the detection of phase-specific gene expression. This will ultimately be done by the functional comparison of identified transcripts in each library and an assessment of their variability in gene expression through microarray analysis or RNA Seq analysis.

### The development of control measures for *O. novo-ulmi*

The multigenic approach to assessing gene expression in *O. novo-ulmi *will also serve the future objective of identifying gene targets that play a key role in the determination of pathogenicity for this species. Such genes will be further studied to assess their potential as targets for biological control strategies. One of the main criteria in the identification of such gene targets will be to confirm that the modification of gene expression at the chosen locus will only induce changes in the fungal species and not in the host, or in other non-target species. As a precursor to this assessment, it will be necessary to compile a prioritized list of possible gene targets identified following the functional characterization of the EST library. A preliminary list of genes has been assembled in the current study and their evaluation can be assisted by the concurrent evaluation of whole organism gene expression made possible by microarray analysis.

The screening of candidate genes is best done by RNA interference (RNAi) as a means of down-regulating the expression of these gene targets. This approach has been used to successfully characterize the role of alpha-(1,3)-glucan synthase (*AGS1*) in the pathogenicity of *H. capsulatum *[42], for the down-regulation of the polyketide synthase (*PKS1*) gene of the melanin pathway in *Ophiostoma piceae *and *Ophiostoma. floccosum *[46] and, more recently, for the evaluation of gene expression by the endopolygalacturonase (*epg1*) gene, a pathogenicity factor in *O. novo-ulmi *[20]. This proven method of gene regulation will provide a means of effectively screening multiple candidate genes from the EST library. Transformed wild type strains of *O. novo-ulmi *with modified expression of selected genes can now be more easily screened in bioassays to assess the impact of targeted RNAi upon strain pathogenicity.

## Conclusions

The creation of an EST library for *O. novo-ulmi *has provided an opportunity for gene discovery and the functional analysis of gene expression in this important plant pathogen. This library will also provide useful information for the study of other *Ophiostoma *spp. of economic importance. A number of genes that may influence virulence and fitness in *O. novo-ulmi *have been identified and these will be the focus of subsequent studies to evaluate their role in host infection. Promising gene targets will be assessed using an RNAi strategy to establish their importance to pathogenicity. These findings will determine the approach of future biological control research to control Dutch elm disease in Canada. This research will be complemented by whole genome expression studies for *O. novo-ulmi *and related species.

## Methods

### Fungal strains and culture conditions

*Ophiostoma novo-ulmi *strain H327, representing a highly aggressive pathogen [47], was selected for RNA extractions. Dimorphic *O. novo-ulmi *can be grown as either a mycelial or a yeast-like form, depending on culture conditions. Stock cultures were maintained on solid *Ophiostoma *complete medium (CM) plates at 23°C [48]. For the generation of yeast-like cultures, 1 cm^2 ^agar plugs were cut from the edge of an actively growing colony, inoculated into a 50 ml volume of liquid CM contained in 125 ml Erlenmeyer flasks [48] and then incubated for 4 days at 23°C with agitation (250 rpm). Yeast cells were subsequently obtained by filtering the liquid culture through 3 layers of sterile miracloth (Calbiotech, La Jolla, CA) and pelleted by centrifugation (700 g) for 15 min.

### Poly(A) mRNA extraction and purification

The extraction and purification of poly(A) RNA was performed using a MicroPoly(A)Pure mRNA Purification Kit (Ambion/Applied Biosystems, Streetsville, ON, Canada). Total RNA was extracted from 210 mg wet weight of yeast cells and the poly (A) RNA was purified by oligo(dT) cellulose spun-column chromatography. The poly (A) RNA was resuspended in 20 μl of RNAase-free sterile, distilled water for storage at -80°C. Spectrophotometric analysis determined the RNA concentration to be 853 ng/μl, with a purity ratio (A_260_/A_280_) of 1.452.

### Complementary DNA synthesis

For construction of the yeast *O. novo-ulmi *cDNA library, the pBluescript II XR cDNA Library Construction kit (Stratagene, La Jolla, CA, USA) was used for the first and second round of cDNA synthesis, cDNA terminus blunting, *Eco*RI adapter ligation and adapter phosphorylation. First-strand synthesis was performed at 42°C for 1 hour with 9.20 μg of the yeast-like mRNA. Samples were cooled on ice for 5 min, prior to second strand synthesis at 16°C for 2.5 hours. The terminus blunting reaction was stopped after 30 min by extraction with 200 μl phenol:chloroform (1:1, v/v). The cDNA with blunt termini were precipitated overnight at -20°C, following the addition of two volumes of 95% ethanol and 0.1 volume of 3 M sodium acetate. The mixture was then centrifuged (13,000 g) for 20 min at 4°C, the supernatant aspirated, the pellet dried by lyophilization and re-suspended in a 9 μl volume containing the *Eco*RI adapters (Stratagene). The adapters were ligated to the blunt cDNA termini, following the addition of 1 μl 10 × ligase buffer, 1 μl 10 mM rATP, 4 units T4 DNA ligase and incubation overnight at 8°C. The ligated *Eco*RI adapters were phosphorylated with 10 units of T4 polynucleotide kinase and digested with 120 units *Xho*I at 37°C for 2 hours. The cDNA was ethanol precipitated overnight at -20°C, centrifuged at 13,000 g for 15 min at 4°C and the pellet was re-suspended in 10 μl Elution Buffer (Qiagen, Mississauga, ON, Canada).

### cDNA size fractionation, ligation and transformation

The synthesized cDNA was size fractionated by electrophoresis on a 1% agarose gel in nuclease-free TAE buffer at 80 V for 1 hour, stained with ethidium bromide and visualized under UV light. The cDNA corresponding to low molecular weight (LMW, 400 - 2,000 bp) and high molecular weight (HMW, 2,000 - 5,000 bp) categories was excised and isolated using the Qiaquick Gel Extraction kit (Qiagen). Fractionated cDNA was eluted with 50 μl Elution Buffer. Spectrophotometric analysis of the isolated yeast HMW and LMW cDNA samples indicated concentrations of 2.8 ng/μl and 3.9 ng/μl, respectively. Fractionated LMW cDNA was ligated into the pBluescript II SK vector (pBluescript II XR cDNA Library Construction kit, Stratagene). Ligation reactions contained 10 ng fractionated cDNA, 20 ng vector, and 2 units of T4 DNA ligase in 1 × ligase buffer with 1 mM rATP (pH 7.5), in a final volume of 5.0 μl that was incubated at 12°C for 24 hours. The resulting constructs were employed to transform ultracompetent *E. coli *DH12S cells by electroporation, using 1 mm gap cuvettes (Bio-Rad, Mississauga, ON, Canada) in a BTX Electro Cell Manipulator 600 (settings: 1.30V; 2.5 kV resistance; capacitance timing = out; 129 Ω). The titer of the transformed bacterial cells was determined by dilution plating on 2YT plates (16 g/L tryptone, 10 g/L yeast extract, 5 g/L NaCl, adjusted to pH 7.0 with 2N NaOH) amended with 50 μg/ml ampicillin (Sigma-Aldrich, Oakville, ON, Canada), 100 μg/ml X-galactose (Sigma), and 31 mg/ml isopropyl β-D-1-thiogalactopyranoside (Sigma). Bacterial titer plates were incubated overnight at 37°C, counted and stored at 4°C for sub-culturing. Plate counts indicated that the yeast LMW library contained approximately 22,000 clones. The primary stock culture of each library was stored at -80°C in 50 μl aliquots to avoid freeze-thaw cycling during sub-culturing.

### DNA sequencing and annotation of ESTs

Clones from the primary yeast LMW cDNA library were prepared for sequencing by plating on 2YT amended with 50 μg/ml ampicillin, at a density of approximately 200 colonies/plate. Discrete colonies were transferred to 96-well cell culture plates (Corning, Lowell, MA, USA) containing 200 μl 2YT amended with 50 μg/ml ampicillin. Cell culture plates were sealed with foil tape (Corning) and incubated overnight at 37°C without shaking. A total of 5,760 clones of the LMW cDNA library were submitted for sequencing and BLASTX analysis.

Downstream processing of the LMW yeast-like *O. novo-ulmi *cDNA library began with the comparison of EST fragments to nucleotide sequences already submitted to public databases. In preparation for sequence comparisons, the vector DNA was edited from authentic *O. novo-ulmi *sequences. Putative identities were assigned to each clone using the heuristic BLASTX algorithm [22], which compares a nucleotide query sequence, translated into all 6 reading frames, against the NCBI Genbank public database. A low-complexity filter was applied to query sequences to remove regions of low-complexity, such as proline-rich regions, or repeats of common acidic or basic residues. The removal of these low-complexity regions increased the fidelity of alignments, and enriched the data for biological significance [49], rather than statistical significance alone.

### Database construction and assignment of functional categories to ESTs

For a list of unique ESTs retrieving hits from public databases, see the Additional File 1 - Alphabetized list of 880 ESTs determined to be unique transcripts with matches to known proteins in the GenBank database). All sequences have been deposited into GenBank's EST database (Accession numbers JG459238 - JG463623).

The Munich Information Centre for Protein Sequences (MIPS, now the Institute for Bioinformatics, Neuherberg, Germany) developed the Functional Catalogue (FunCat) as a stand-alone information management framework and it has become a standard tool for bioinformatics studies [25,50]. FunCat is a hierarchically structured, scalable classification system enabling the functional assignment of proteins from any genome according to their physiological role, or metabolic pathway.

A transcription profile was created for the *O. novo-ulmi *Yeast LMW library using transcripts which matched sequences characterized in other organisms. These were subjected to further BLAST analysis to obtain the three highest scoring alignments and this information was manually scrutinized to determine the most meaningful annotation for each EST within the FunCat scheme. It is important to note that many proteins are associated with more than one metabolic pathway and many pathways influence more than one aspect of metabolism. Consequently, the assignment of a single functional category to a protein can be both restrictive and inaccurate. Many multifunctional proteins are justifiably included in numerous functional categories. This can result in a small number of proteins generating a very large number of functional assignments. In order to standardize FunCat scores for the Yeast LMW EST library and accommodate multifunctional proteins, we assigned each protein a total of 1.00 units of metabolic function, such that multifunctional proteins were assigned a value less than one, as dictated by the number of functional categories they encompassed [metabolic function = x(1/x), where × = number of functional categories included and 1/x = proportion of metabolic function assigned to each category].

## Authors' contributions

WH was the supervisor for the project, conducted data analysis and database creation and was responsible for the preparation and submission of the manuscript. CB and LB were co-Principal Investigators on this project and assisted with data analysis as well as providing input to the manuscript. The experimental work was conducted by SB, MP and PB. Additional data analysis and revision of the manuscript was done by VJ and RH. All authors have read and approved the final manuscript.

## Supplementary Material

Additional file 1 **Identified genes and FunCat assignments**.xls (excel spreadsheet). Click here for file
